# Treatment of FIGO 2018 stage IIIC cervical cancer with different local tumor factors

**DOI:** 10.1186/s12885-023-10801-w

**Published:** 2023-05-09

**Authors:** Yanna Ye, Zhiqiang Li, Shan Kang, Yongxiu Yang, Bin Ling, Li Wang, Jilong Yao, Pengfei Li, Xueqin Wang, Shipeng Gong, Huijian Fan, Yanxiang Kong, Yuye Cao, Jinghe Lang, Ping Liu, Chunlin Chen

**Affiliations:** 1 Department of Obstetrics and Gynecology, Nanfang Hospital, Southern Medical University, No. 1838, Guangzhou Avenue, Guangzhou, 510515 China; 2 Department of Midwifery, Faculty of Health, Dongguan Polytechnic, Dongguan, 523000 China; 3 Department of Gynecology, Fourth HospitalHebei Medical University, Shijiazhuang, 050019 China; 4 Department of Obstetrics and Gynecology, First Hospital, Lanzhou University, Lanzhou, 730000 China; 5 Department of Obstetrics and Gynecology, China-Japan Friendship Hospital, Beijing, 100029 China; 6 Department of Gynecologic Oncology, Affiliated Cancer Hospital, Zhengzhou University, Zhengzhou, 450008 China; 7 Shenzhen Maternal and Child Health Hospital, Shenzhen, 518028 China; 8 Department of Obstetrics and Gynecology, The Fifth Affiliated Hospital, Southern Medical University, Guangzhou, 510920 China; 9 The Third Affiliated Hospital, Guangzhou Medical University, Guangzhou, 510150 China; 10 Reproductive Medicine Center, The Seventh Affiliated Hospital, Sun Yat-Sen University, Shenzhen, 518107 China; 11 Department of Obstetrics and Gynecology, Peking Union Medical College Hospital, Beijing, 100193 China

**Keywords:** Cervical cancer, FIGO 2018 stage IIIC, Local tumor factors, Treatment

## Abstract

**Background:**

To compare the oncological outcomes of patients with FIGO 2018 stage IIIC cervical cancer (CC) involving different local tumor factors who underwent abdominal radical hysterectomy (ARH), neoadjuvant chemotherapy and radical surgery (NACT), or radical chemoradiotherapy (R-CT).

**Methods:**

Based on tumor staging, patients with stage IIIC were divided into T1, T2a, T2b, and T3 groups. Kaplan–Meier and Cox proportional hazards regression analysis were used to compare their overall survival (OS) and disease-free survival (DFS) of 5 years.

**Results:**

We included 4,086 patients (1,117, 1,019, 869, and 1,081 in the T1, T2a, T2b, and T3 groups, respectively). In the T1 group, NACT was correlated with a decrease in OS (hazard ratio [HR] = 1.631, 95% confidence interval [CI]: 1.150–2.315, *P* = 0.006) and DFS (HR = 1.665, 95% CI: 1.255–2.182, *P* < 0.001) than ARH. ARH and NACT were not correlated with OS (*P* = 0.226 and *P* = 0.921) or DFS (*P* = 0.343 and *P* = 0.535) than R-CT. In the T2a group, NACT was correlated with a decrease in OS (HR = 1.454, 95% CI: 1.057–2.000, *P* = 0.021) and DFS (HR = 1.529, 95% CI: 1.185–1.974, *P* = 0.001) than ARH. ARH and NACT were not correlated with OS (*P* = 0.736 and *P* = 0.267) or DFS (*P* = 0.714 and *P* = 0.087) than R-CT. In the T2b group, NACT was correlated with a decrease in DFS (HR = 1.847, 95% CI: 1.347–2.532, *P* < 0.001) than R-CT nevertheless was not correlated with OS (*P* = 0.146); ARH was not correlated with OS (*P* = 0.056) and DFS (*P* = 0.676). In the T3 group, the OS rates of ARH (*n* = 10), NACT (*n* = 18), and R-CT (*n* = 1053) were 67.5%, 53.1%, and 64.7% (*P* = 0.941), and the DFS rates were 68.6%, 45.5%, and 61.1%, respectively (*P* = 0.761).

**Conclusion:**

R-CT oncological outcomes were not entirely superior to those of NACT or ARH under different local tumor factors with stage IIIC. NACT is not suitable for stage T1, T2a, and T2b. Nevertheless ARH is potentially applicable to stage T1, T2a, T2b and T3. The results of stage T3 require confirmation through further research due to disparity in case numbers in each subgroup.

**Supplementary Information:**

The online version contains supplementary material available at 10.1186/s12885-023-10801-w.

## Background

CC is the predominant genital-tract malignancy in women. Tumor staging reflects both prognosis and survival, and is crucial for the management of tumor and treatment guidance. International Federation of Gynecology and Obstetrics (FIGO) staging is widely used for CC. In 2018, FIGO released a new staging process [1], which was revised in 2019 [2]. It was mainly updated to add stage IIIC, referring to involvement of the pelvic lymph nodes(LNs) and/or para-aortic LNs regardless of tumor size and extent of spread. Certain scholars dispute stage IIIC prognosis, which is solely based on the lymph-node-metastasis stage, without considering the local invasion scope, tumor size, and other factors [3]. The “2022 NCCN Cervical Cancer Clinical Practice Guidelines (1st Edition)” recommend R-CT for stage IIIC [4]; therefore, patients with relatively localized tumors are denied surgery.

Although FIGO staging is recommended by most medical guidelines, the European Society of Gynecologic Oncology, European Society of Radiotherapy and Oncology, and European Society of Pathology guidelines recommend tumor-node-metastasis (TNM) staging. Therefore, certain scholars have raised the following question: “Using T-staging, are the oncological outcomes of different treatments in patients with FIGO 2018 stage IIIC consistent?” To date, only a small number of studies have focused on this stage, using small sample sizes and few stratified comparisons. Therefore, based on the 1538 project database, our study screened FIGO 2018 stage IIIC cases using T-staging stratification, that is, according to T1, T2a, T2b, and T3 stratification (corresponding to FIGO 2009 stages I, IIA, IIB, and III cases with LN metastasis), to investigate the survival outcomes of ARH, NACT, and R-CT for the selection of appropriate treatment strategies for FIGO 2018 stage IIIC CC.

## Methods

### Data collection

Chinese Cervical Cancer Clinical (Four-C) study is a multi-center, retrospective cohort study, including 63,926 patients with various stages of CC who were hospitalized in 47 Chinese hospitals during 2004–2018. This study was approved by the Ethics Committee of Nanfang Hospital, Southern Medical University (ethical number: NFEC-2017–135, Clinical trial Registration Number: CHiCTR1800017778). We collect the following data by reviewing electronic medical records: general clinical data, preoperative laboratory test results, preoperative pathological results, relevant surgical data, preoperative adjuvant treatment data, postoperative adjuvant therapy data, postoperative pathological results, and follow-up data. We phoned to follow up and obtained messages of survival, recurrence, and complications, if failed, we obtained information from inpatient and outpatient medical records. All original data were reviewed and validated by two independent gynecologists to ensure the accuracy. The details of case collection in the Four-C study database is as shown in our previous studies [5–7]. In accordance with the journal’s guidelines, we will provide our data for the reproducibility of this study in other centers if such is requested.

### Inclusion and exclusion criteria

TNM-staging tumor factors [8] were used to divide IIIC-stage patients into four groups: T1, T2a, T2b, and T3.

The following criteria were for inclusion: age ≥ 18 years; CC diagnosed by cervical biopsy; squamous cell carcinoma(SCC), adenocarcinoma(AC), or adenosquamous cell carcinoma(ASC) based on histology; FIGO 2018 stage IIIC (patients who underwent radiation therapy defined lymph node status depended on CT, MRI or/and PET-CT before treatment, and those who underwent radical surgery defined lymph node status depended on pathological examinations after surgery); ARH-group patients who underwent radical surgery (Q-M type-B or type-C radical hysterectomy pelvic lymphadenectomy ± para-aortic lymphadenectomy); NACT-group patients who underwent neoadjuvant therapy and radical surgery (Q-M type-B or type-C radical hysterectomy pelvic lymphadenectomy ± para-aortic lymphadenectomy); R-CT-group patients who underwent radiation therapy(RT),and RT dose ≥ 45 Gy; with follow-up outcomes.

The following criteria were for exclusion: gestation, cervical stump cancer, CC complicated with other malignant tumors, loss to follow-up, and failure to satisfy the inclusion criteria.

### Outcome measurement

The main outcome measures were overall survival (OS) and disease-free survival (DFS) in all subgroups of FIGO 2018 stage IIIC, with the cut-off point 5 years after treatment. The concept of OS is the final time from diagnosis to effective follow-up or death from any cause, moreover the concept of DFS is the final time from diagnosis to effective follow-up, recurrence or death.

### Statistics

No data were missing among the included cases. Mean ± standard deviation (x ± s) was used to represent continuous data, furthermore percentage (%) was used to represent counting data. Fisher's exact test or Chi-square test were used to compare categorical variables. Kaplan–Meier curves were used to describe changes in survival outcomes. Cox proportional risk regression models were used to adjust variables and evaluate the HRs and 95% CI of stratification for 5-year OS and DFS. SPSS 26.0 (SPSS, Inc., Chicago, IL, USA) was used for statistical analyses, and statistical significance was set at *P* < 0.05.

## Results

**Fig. 1:**
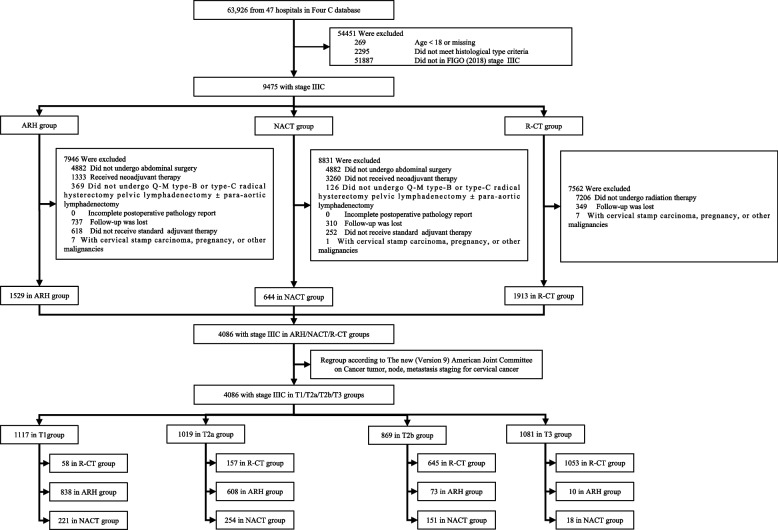
Flow diagram of recruitment and exclusion

Based on the inclusion criteria, 4,086 CC cases (including 1,117, 1,019, 869, and 1,081 cases in the T1, T2a, T2b, and T3 groups, respectively) were included. Data screening process is shown in Fig. 1.

### Clinicopathological characteristics of each group

Clinicopathological characteristics of each group are shown in Table 1. Clear distinctions were shown among the median age of the patients using the three treatments in the T1, T2a, T2b, and T3 groups (*P* < 0.05 for all). No sharp distinctions were observed in the proportion of histologic types between the T1 and T2a groups (*P* > 0.05 for all); however, a statistically significant distinction was shown between the T2b and T3 groups (*P* < 0.001 for all).

**Table 1:** Clinicopathological characteristics of patients of T1, T2a, T2b and T3 group

Characteristics	T1 group (*n* = 1117)				T2a group (*n* = 1019)				T2b group (*n* = 869)				T3 group (*n* = 1081)			
	R-CT	ARH	NACT	*P*	R-CT	ARH	NACT	*P*	R-CT	ARH	NACT	*P*	R-CT	ARH	NACT	*P*
	(n) %	(n) %	(n) %		(n) %	(n) %	(n) %		(n) %	(n) %	(n) %		(n) %	(n) %	(n) %	
Age(years)	53.83 ± 12.70	46.78 ± 9.04	44.72 ± 8.25	< 0.001	55.01 ± 10.48	49.98 ± 9.54	47.62 ± 8.34	< 0.001	54.44 ± 10.78	48.93 ± 9.81	47.55 ± 8.41	< 0.001	54.64 ± 10.8	54.9 ± 9.01	48.33 ± 9.02	0.048
Histological type				0.082				0.141				< 0.001				< 0.001
Squamous cell carcinoma	57(98.3)	727(86.8)	192(86.9)		148(94.3)	552(90.8)	226(89.0)		624(96.7)	67(91.8)	130(86.1)		1014(96.3%)	9(90.0%)	15(83.3%)	
Adenocarcinoma	0(0)	76(9.1)	23(10.4)		3(1.9)	40(6.6)	20(7.9)		17(2.6)	6(8.2)	13(8.6)		30(2.8%)	1(10%)	1(5.6%)	
Adenosquamous cell carcinoma	1(1.7)	35(4.2)	6(2.7)		6(3.8)	16(2.6)	8(3.1)		4(0.6)	0(0)	8(5.3)		9(0.9%)	0(0%)	2(11.1%)	

### Comparison of oncological outcomes of the three treatment methods in each group

#### Comparison of oncological outcomes of the three treatment methods in the T1 group

**Fig. 2:**
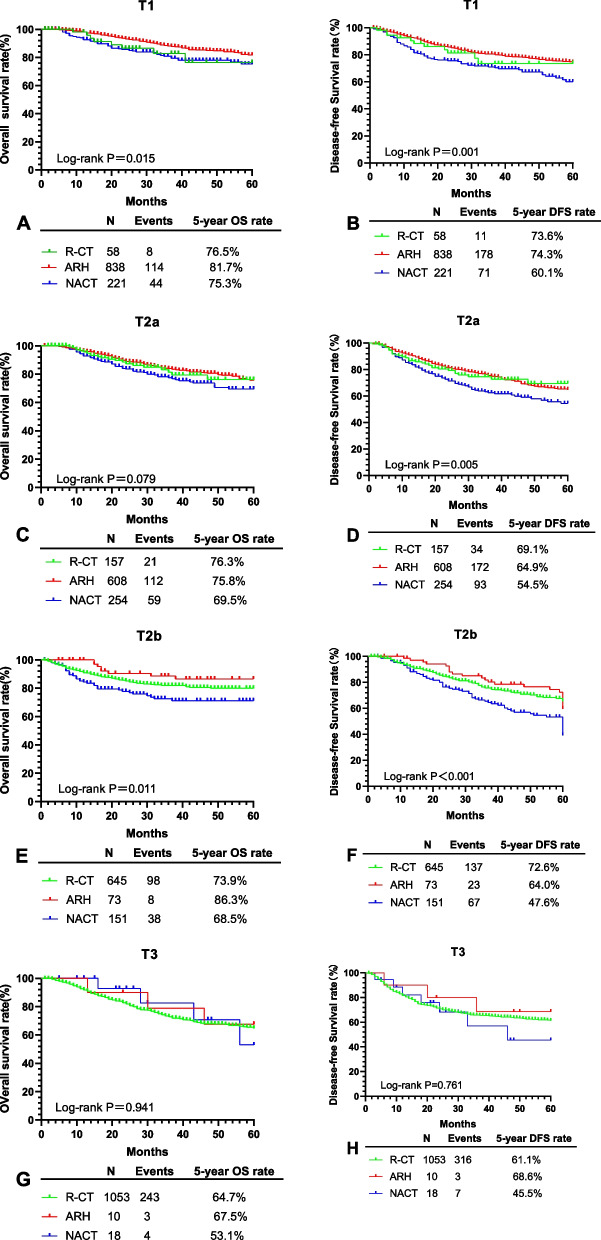
OS and DFS of three treatment in T1, T2a, T2b and T3 group

In the T1 group, 1,117 cases were divided into R-CT (*n* = 58), ARH (*n* = 838), and NACT (*n* = 221) subgroups. Kaplan–Meier analysis revealed statistically sharp distinctions in 5-year OS (76.5% *vs*. 81.7% *vs*. 75.3%, *P* = 0.015) and DFS ( 73.6% *vs*.74.3% *vs*. 60.1%, *P* = 0.001) among the three subgroups (Fig. 2A, B). Cox proportional hazards regression analysis revealed NACT was not correlated with 5-year OS (HR = 1.040, 95% CI: 0.481–2.249, *P* = 0.921) or DFS (HR = 1.227, 95% CI: 0.642–2.344, *P* = 0.535) than R-CT. Furthermore, ARH was not correlated with 5-year OS (HR = 0.637, 95% CI: 0.307–1.322, *P* = 0.226) and 5-year DFS (HR = 0.741, 95% CI: 0.400–1.375, *P* = 0.343) than R-CT (Table 2). However, NACT was correlated with a decrease in OS (HR = 1.631, 95% CI: 1.150–2.315, *P* = 0.006) and DFS (HR = 1.665, 95% CI: 1.255–2.182, *P* < 0.001) than ARH (Table 3). AC was associated with a decrease in 5-year OS (HR = 1.734, 95% CI: 1.092–2.573, *P* = 0.020) and 5-year DFS (HR = 1.786, 95% CI: 1.241–2.570, *P* = 0.002) than SCC; nonetheless, ASC was not correlated with 5-year OS and DFS (*P* > 0.05 for all). Moreover, age was not correlated with 5-year OS and DFS (*P* > 0.05 for all) (Tables 2 and 3).

**Table 2:** Cox multivariate survival analysis for T1 and T2a group

Variables	T1 group (*n* = 1117)						T2a group (*n* = 1019)					
	5-year OS			5-year DFS			5-year OS			5-year DFS		
	HR	95%CI	*P*	HR	95%CI	*P*	HR	95%CI	*P*	HR	95%CI	*P*
Age(years)	1.008	0.992–1.026	0.327	0.996	0.983–1.010	0.599	1.008	0.993–1.024	0.311	1.005	0.993–1.018	0.398
Histological type			0.061			0.008			< 0.001			0.011
Squamous cell carcinoma	1	-	-	1	-	-	1	-	-	1	-	-
Adenocarcinoma	1.734	1.092–2.753	0.020	1.786	1.241–2.570	0.002	1.613	0.949–2.743	0.078	1.273	0.806–2.008	0.3
Adenosquamous cell carcinoma	1.267	0.559–2.874	0.571	1.041	0.513–2.110	0.912	3.182	1.803–5.617	< 0.001	2.21	1.288–3.792	0.004
initiatreatment modality			0.016			0.001			0.070			0.005
R-CT	1	-	-	1	-	-	1	-	-	1	-	-
ARH	0.637	0.307–1.322	0.226	0.741	0.400–1.375	0.343	0.921	0.572–1.484	0.736	0.932	0.640–1.357	0.714
NACT	1.040	0.481–2.249	0.921	1.227	0.642–2.344	0.535	1.339	0.800–2.243	0.267	1.425	0.950–2.139	0.087

**Table 3:** Cox multivariate survival analysis for groups (T1 and T2a)

Variables	T1 group (*n* = 1117)						T2a group (*n* = 1019)					
	5-year OS			5-year DFS			5-year OS			5-year DFS		
	HR	95%CI	*P*	HR	95%CI	*P*	HR	95%CI	*P*	HR	95%CI	*P*
Age(years)	1.008	0.992–1.026	0.327	0.996	0.983–1.010	0.599	1.008	0.993–1.024	0.311	1.005	0.993–1.018	0.398
Histological type			0.061			0.008			< 0.001			0.011
Squamous cell carcinoma	1	-	-	1	-	-	1	-	-	1	-	-
Adenocarcinoma	1.734	1.092–2.753	0.020	1.786	1.241–2.570	0.002	1.613	0.949–2.743	0.078	1.273	0.806–2.008	0.300
Adenosquamous cell carcinoma	1.267	0.559–2.874	0.571	1.041	0.513–2.110	0.912	3.182	1.803–5.617	< 0.001	2.21	1.288–3.792	0.004
initiatreatment modality			0.016			0.001			0.070			0.005
ARH	1	-	-	1	-	-	1	-	-	1	-	-
R-CT	1.569	0.756–3.254	0.226	1.349	0.727–2.501	0.343	1.085	0.674–1.748	0.736	1.073	0.737–1.562	0.714
NACT	1.631	1.150–2.315	0.006	1.655	1.255–2.182	< 0.001	1.454	1.057–2.000	0.021	1.529	1.185–1.974	0.001

#### Comparison of oncological outcomes of the three treatments in the T2a group

In the T2a group, 1,019 cases were divided into R-CT (*n* = 157), ARH (*n* = 608), and NACT (*n* = 254) subgroups. Kaplan–Meier analysis revealed statistically sharp distinctions in 5-year DFS (69.1% *vs*. 64.9% *vs*. 54.5%, *P* = 0.005) among the three subgroups; however, there was no distinctions in 5-year OS ( 76.3% *vs*. 75.8% *vs*. 69.5%, *P* = 0.079) (Fig. 2C, D). Cox proportional hazards regression analysis revealed NACT was not correlated with 5-year OS (HR = 1.339,95% CI:0.800–2.243, *P* = 0.267) or DFS (HR = 1.425,95% CI:0.950–2.139, *P* = 0.087) than R-CT. Furthermore, ARH was not correlated with 5-year OS (HR = 0.921, 95% CI: 0.572–1.484, *P* = 0.736) and 5-year DFS (HR = 0.932, 95% CI: 0.640–1.357, *P* = 0.714) than R-CT (Table 2). Nevertheless, NACT was correlated with a decrease in OS (HR = 1.454, 95% CI: 1.057–2.000, *P* = 0.021) and DFS (HR = 1.529, 95% CI: 1.185–1.974, *P* = 0.001) than ARH (Table 3). ASC was associated with a decrease in 5-year OS (HR = 3.182, 95% CI: 1.803–5.617, *P* < 0.001) and 5-year DFS (HR = 2.210, 95% CI: 1.288–3.792, *P* = 0.004) than SCC; nonetheless, AC was not correlated with 5-year OS and DFS (*P* > 0.05 for all). Moreover, age was not associated with 5-year OS and DFS (*P* > 0.05 for all) (Tables 2 and 3).

#### Comparison of the oncological outcomes of the three treatments in the T2b group

In the T2b group, 869 cases were divided into R-CT (*n* = 645), ARH (*n* = 73), and NACT (*n* = 151) subgroups. Kaplan–Meier analysis revealed statistically sharp distinctions in 5-year OS (73.9% *vs*. 86.3% *vs*. 68.5%, *P* = 0.011) and DFS (72.6% *vs*. 64.0% *vs*. 47.6%, *P* < 0.001) among the three subgroups (Fig. 2E, F). Cox proportional hazards regression analysis revealed NACT correlated with a decrease in 5-year DFS (HR = 1.847, 95% CI: 1.347–2.532, *P* < 0.001) than R-CT; however, there was no correlation with OS (HR = 1.353, 95% CI: 0.901–2.032, *P* = 0.146). ARH was not correlated with 5-year OS (HR = 0.490, 95% CI: 0.236–1.017, *P* = 0.056) and DFS (HR = 1.101, 95% CI 0.702–1.725, *P* = 0.676). AC was correlated with a decrease in 5-year OS (HR = 1.999, 95% CI: 1.092–3.660, *P* = 0.025) and DFS (HR = 2.098, 95% CI: 1.298–3.392, *P* = 0.003) than SCC. However, ASC was not correlated with 5-year OS and DFS (*P* > 0.05 for all). Furthermore, age was not correlated with 5-year OS and DFS (*P* > 0.05 for all) (Table 4).

**Table 4:** Cox multivariate survival analysis for T2b and T3 group

Variables	T2b group (*n* = 869)						T3 group (*n* = 1081)					
	5-year OS			5-year DFS			5-year OS			5-year DFS		
	HR	95%CI	*P*	HR	95%CI	*P*	HR	95%CI	*P*	HR	95%CI	*P*
Age(years)	1.007	0.991–1.024	0.387	0.997	0.984–1.010	0.644	1.004	0.993–1.016	0.464	0.999	0.989–1.009	0.840
Histological type			0.022			0.001			0.035			0.110
Squamous cell carcinoma	1	-	-	1	-	-	1	-	-	1	-	-
Adenocarcinoma	1.999	1.092–3.660	0.025	2.098	1.298–3.392	0.003	1.605	0.851–3.027	1.144	1.405	0.769–2.567	0.269
Adenosquamous cell carcinoma	2.33	0.993–5.817	0.070	1.623	0.711–3.705	0.250	2.733	1.109–6.733	0.029	2.156	0.946–4.916	0.068
initiatreatment modality			0.030			0.003			0.870			0.865
R-CT	1	-	-	1	-	-	1	-	-	1	-	-
ARH	0.490	0.236–1.017	0.056	1.101	0.702–1.725	0.676	0.879	0.281–2.746	0.824	0.747	0.239–2.328	0.614
NACT	1.353	0.901–2.032	0.146	1.847	1.347–2.532	< 0.001	0.780	0.284–2.139	0.629	1.075	0.499–2.316	0.853

#### Comparison of the oncological outcomes of the three treatments in the T3 group

In the T3 group, 1,081 cases were divided into R-CT (*n* = 1,053), ARH (*n* = 10), and NACT (*n* = 18) subgroups. Kaplan–Meier analysis revealed no statistically sharp distinctions in 5-year OS (64.7% *vs*. 67.5% *vs*. 53.1%, *P* = 0.941) and DFS (61.1% *vs*. 68.6% *vs*. 45.5%, *P* = 0.761) among the three subgroups (Fig. 2G, H). Cox proportional hazards regression analysis revealed NACT was not correlated with 5-year OS (HR = 0.780, 95% CI: 0.284–2.139, *P* = 0.629) and DFS (HR = 1.075, 95% CI: 0.499–2.316, *P* = 0.853) compared with R-CT. ARH was not correlated with 5-year OS (HR = 0.879, 95% CI: 0.281–2.746, *P* = 0.824) and DFS (HR = 0.747, 95% CI: 0.239–2.328, *P* = 0.614). ASC was correlated with a decrease in 5-year OS (HR = 2.733, 95% CI: 1.109–6.733, *P* = 0.029) than SCC; nevertheless, it was not correlated with 5-year DFS (HR = 2.156, 95% CI: 0.946–4.916, *P* = 0.068), and AC was not correlated with 5-year OS and DFS (*P* > 0.05 for all). In addition, age was not correlated with 5-year OS and DFS (*P* > 0.05 for all) (Table 4).

## Discussion

### Summary of main results

A key change to the FIGO 2018 staging system was LN metastasis inclusion, which indicates its importance in tumor progression and prognosis. Thus, this group is treated differently, which is helpful for clinical research and medical intervention. However, the 2018 FIGO Cervical Cancer guidelines have no stratified treatment recommendations for these patients. Since the release of the 2018 FIGO staging system, a wave of research on new staging treatment strategies and prognosis has emerged. Based on the 1538 project database, this study conducted a real-world study on FIGO 2018 stage IIIC CC using T-staging to investigate the oncological outcomes of ARH, NACT, and R-CT. The results demonstrate that different treatments affect the oncological outcomes of patients with T1, T2a, T2b, and T3 CC in FIGO 2018 stage IIIC. Mortality and recurrence/death risks were higher in NACT than in ARH in the T1 and T2a group, while recurrence/death risk was higher in NACT than in R-CT in the T2b group. Among the T1, T2a, T2b, and T3 groups, no statistically significant differences in death and recurrence/death risks were noted between ARH and R-CT. ARH may be an alternative initial treatment for patients with FIGO 2018 stage IIIC CC. NACT is not recommended for stage T1, T2a and T2b. The number of cases in the three subgroups of the T3 stage varied greatly, therefore further research is required to confirm the results.

### Results in the context of published literature

#### TNM-staging

Valid staging systems are characterized by intragroup homogeneity, that is, same-staged patients essentially exhibit minimal prognostic differences. Previous related studies suggested that although LN metastasis is an important prognostic factor for patients with CC, including all patients with LN metastasis in the same stage leads to high patient heterogeneity [9, 10]. Certain studies found that CC prognosis was related to T-staging [11, 12]; these results may be related to stage IIIC patient heterogeneity. In a retrospective study by Matsuo K et al. [9], 733 patients with stage IIIC1 were divided into T1, T2, and T3 groups based on T-staging. T3b had a lower survival rate, revealing a significant OS difference based on T-staging among stage IIIC1 patients; however, this study exclusively included stage IIIC1 patients. Evidently, the prognosis of stage IIIC patients is also affected by local tumor factors, with a significantly different OS rate. The vast heterogeneity among these patients not only affects prognostic prediction but also clinical decision-making. Treating stage IIIC patients, with sole consideration of LN status, without stratifying local tumor factors and extent of spread, may be inappropriate. Therefore, our study stratified stage IIIC cases using T-staging and compared the oncological outcomes of ARH, NACT and R-CT.

#### Treatment strategies

CC treatment should be implemented in a planned and sequential stage-based manner, adjusting according to surgical results and post-radiotherapy tumor regression. Due to the advantages of preserving ovarian function, tissue elasticity, and reproductive function, surgical-treatment is increasing [13]. RT is predominant for advanced cervical cancer. Chemotherapy is used as an adjuvant therapy for RT sensitization.

Radical surgical treatment can remove metastatic pelvic LN to minish burthen of tumor and determine LN status, thus guiding postoperative supplementary treatment selection. Avoiding excessive treatment is important. Currently, radical surgery and pelvic lymphadenectomy are preferred for early CC. A study of FIGO 2009 IB1 and IIA1 CC by Wu et al. [14] suggested that no significant oncological-outcome difference existed between ARH and R-CT. Landoni’s study of 19/343 IB1 and IIA1 CC cases also concurred that ARH and R-CT had similar effects. Our study agreed with these findings. However, a study on FIGO 2009 IB1-IIA2 CC [15] concluded that ARH oncological outcomes were superior to those of R-CT; nevertheless, it exclusively included SCC cases. A seven-study meta-analysis by Yan et al. [16] revealed that ARH had obvious advantages over chemoradiotherapy for IB2-IIA CC. Jang et al. [17] conducted a study on FIGO 2009 IB1-IIA CC and found that the oncological prognosis of ARH was significantly superior to that of concurrent chemoradiotherapy (CCRT). Bansal et al. [18] analyzed 4,885 cases of FIGO 2009 IB1-IIA CC and found that when the tumor diameter is < 6 cm, ARH potentially benefits patient survival more than R-CT; when it is > 6 cm, the two are equivalent. For FIGO 2018 stage IIIC, the oncological prognosis of R-CT does not prevail over that of ARH, considering that R-CT has a series of complications, such as weakened ovarian function [19], vaginal constriction, and dry intercourse [20], which seriously affect patients’ quality of life. Hence, ARH is recommended as an initial treatment.

NACT is the predominant pre-operative adjuvant therapy for CC, while FIGO guidelines recommend it for clinical trials or areas where radiotherapy equipment is lacking. NCCN guidelines only recommend it for small-cell neuroendocrine carcinoma of the cervix. At present, whether NACT can improve the prognosis of patients with CC remains controversial [21, 22]. In the T1 and T2a group of ours, NACT was associated with a decrease in 5-year OS and 5-year DFS than ARH. Meantime, relevant studies found worse prognosis or no difference between post-NACT surgery and radical radiotherapy for patients with CC. A single-center, phase III, randomized controlled trial by Gupta et al. [23] found that for FIGO 2009 stage IB2-IIB cervical SCC, the group undergoing post-NACT surgery had a statistically difference in 5-year DFS compared with the CCRT group; however no sharp distinction in 5-year OS. In the T2b group of ours, NACT was correlated with a decrease in 5-year DFS than R-CT; nevertheless, it was not correlated with 5-year OS. Duenas-Gonzalez et al. [24] did not detect any difference in response and viability between post-chemotherapy surgery and standard cisplatin-based chemoradiotherapy. Similar results were obtained in the T1, T2a and T3 groups in ours, and no statistical distinction in 5-year OS and DFS was noted between NACT and R-CT. Evidently, in FIGO 2018 stage IIIC, patients in the T1 and T2a groups potentially benefited more from ARH than NACT; meanwhile, in the T2b group, NACT was not efficaciously advantageous. Therefore, NACT should be used with caution in stage T1,T2a and T2b.

Previous studies predominantly used FIGO 2009 staging, included cases with positive and negative LN, and did not exclude patients treated with laparoscopic surgery. Differences in adjuvant-treatment regimens and treatment courses also existed. Several studies advocate that LN metastasis is an important factor affecting CC prognosis [25–28]. Results of LACC study in 2018 [29] suggested that laparoscopic surgery had adverse oncology outcomes in patients with CC. Therefore, ours only included LN-positive cases and was limited to laparotomy-treated patients, thus potentially justifying the inconsistencies between this and previous studies.

### Strengths and weaknesses

Ours is a large-scale research based on FIGO 2018 stage IIIC CC cases. Notably, it innovatively stratified patients basing on local tumor conditions to compare the prognosis of different treatment methods. Notwithstanding, this study also had some shortcomings. First, this was a real-world, retrospective analysis, resulting in unbalanced data between groups. Second, the numbers of cases among the T3 subgroup were significantly different, thus potentially affecting the results’ reliability. Third, there is a limitation on a distinction between staging modalities. Patients who underwent radical surgery in the ARH and NACT groups defined LN status depended on pathological examinations after surgery; nevertheless, those who underwent radical chemoradiotherapy in the R-CT group defined lymph node status depended on CT or/and MRI before surgery. There is a difference in false positive rates between the two methods. Fourth, no further stratification based on N stage was conducted in this study due to insufficient information on the status of para-aortic lymph nodes.

### Implications for practice and future research

In conclusion, for patients with FIGO 2018 stage IIIC CC, different treatment strategies impact oncological outcomes. When selecting a treatment strategy for these patients, T-staging is required. Patients with stages T1, T2a, T2b, and T3 can select ARH for initial treatment. Because of the huge differences in the number of cases among the T3 subgroups, our results require confirmation through further research. NACT is not recommended for patients with stage T1, T2a and T2b. Evidently, the recommended treatment methods for patients with stage IIIC CC in the guidelines are debatable, and more prospective studies are warranted.

## Conclusions

R-CT oncological outcomes were not entirely superior to those of NACT or ARH under different local tumor factors with stage IIIC. NACT is not suitable for stage T1, T2a and T2b. Nevertheless ARH is potentially applicable to stage T1, T2a, T2b and T3. Stage T3 results require confirmation through further research due to disparity in case numbers in each subgroup.

## Supplementary Information

**Additional file 1.**

## Data Availability

The datasets used and/or analyzed for the current study are available from the corresponding author upon reasonable request.

## Abbreviations

CC: Cervical cancer
FIGO: International Federation of Gynecology and Obstetrics
NCCN: National Comprehensive Cancer Network
ARH: Abdominal radical hysterectomy
TNM: Tumor-node-metastasis
NACT: Neoadjuvant chemotherapy
R-CT: Radical chemoradiotherapy
SCC: Squamous cell carcinoma
ASC: Adenosquamous cell carcinoma
OS: Overall survival
DFS: Disease-free survival
AC: Adenocarcinoma
