# Supplementary material for: Treatment of FIGO 2018 stage IIIC cervical cancer with different local tumor factors
Source: BMC Cancer. 2023 May 9;23:421. doi: 10.1186/s12885-023-10801-w (PMC10170857; doi:10.1186/s12885-023-10801-w)
Supplement: Supplementary file 1 — Additional file 1. [file 12885_2023_10801_MOESM1_ESM.docx]

**Treatment of FIGO 2018 stage IIIC cervical cancer with different local tumor factors**

**Highlights:**

- R-CT oncological outcomes are not superior to those of NACT or ARH.
- NACT is not suitable for stage T1, T2a, and T2b.
- ARH is potentially applicable to stage T1, T2a, T2b and T3.
